# Struma ovarii presenting with Hashimoto's thyroiditis: a case report

**DOI:** 10.1186/1752-1947-5-572

**Published:** 2011-12-12

**Authors:** Nujen Çolak Bozkurt, Başak Karbek, Evrim Çakır Özkaya, Erman Çakal, Tuncay Delibaşi

**Affiliations:** 1 Department of Endocrinology and Metabolism, Ankara Diskapi Training and Research Hospital, 781/2 Sok. No:1 Altindag Caddesi, Yildirim Beyazit Mahallesi, 06115 Altindag- Ankara, Turkey

## Abstract

**Introduction:**

We report the case of a patient diagnosed with a struma ovarii with lymphocytic thyroiditis of her ectopic thyroid tissue. We believe that this case presents an unusual variation of a struma ovarii and a rare presentation of subclinical hyperthyroidism.

**Case presentation:**

A 17-year-old Caucasian female patient who had undergone an ovariectomy and been diagnosed with a struma ovarii was subsequently found to have persistent subclinical hyperthyroidism with a low radioiodine uptake. Abdominal magnetic resonance imaging and iodine-131 whole body scanning showed no residue or recurrence and a thyroid ultrasonography was normal. Laboratory and histopathological findings suggested Hashimoto's thyroiditis as the cause of the subclinical thyrotoxicosis, which had presumably started at the ectopic tissue.

**Conclusion:**

Struma ovarii is a rare cause of thyrotoxicosis, and can be difficult to diagnose in the presence of co-existing thyroid disorders. In patients with a struma ovarii who have not undergone thyroidectomy, there is no common consensus on management in terms of residue, recurrence or metastasis. Autoimmune thyroiditis must be kept in mind for a differential diagnosis.

## Introduction

A struma ovarii is an unusual type of mature teratoma consisting of thyroid epithelium. It shows mostly benign histopathological features of thyroid tissue [1]. Hyperthyroidism develops in approximately 5% to 15% of patients, mostly due to an adenoma, and rarely due to follicular carcinoma [2]. However autoimmune thyroiditis with a struma ovarii has been described in a few case reports. A struma ovarii generally presents with non-specific symptoms that are similar to those of other ovarian neoplasms. Diagnosis is difficult unless the tumor is very large or causes remarkable thyrotoxicosis. Radioiodine (I-131) imaging and thyroid uptake studies are important for the differential diagnosis of thyrotoxicosis, which can be difficult when a struma ovarii is also present [3]. The appropriate follow-up of patients with a struma ovarii in terms of residue, recurrence or metastasis after surgical resection is a current topic of debate, especially for patients with co-existing thyroid disorders. Here, we report a patient who underwent an ovariectomy and was diagnosed with a struma ovarii, and subsequently was found to have persistent subclinical hyperthyroidism as a manifestation of Hashimoto's thyroiditis.

## Case presentation

**Figure 1:**
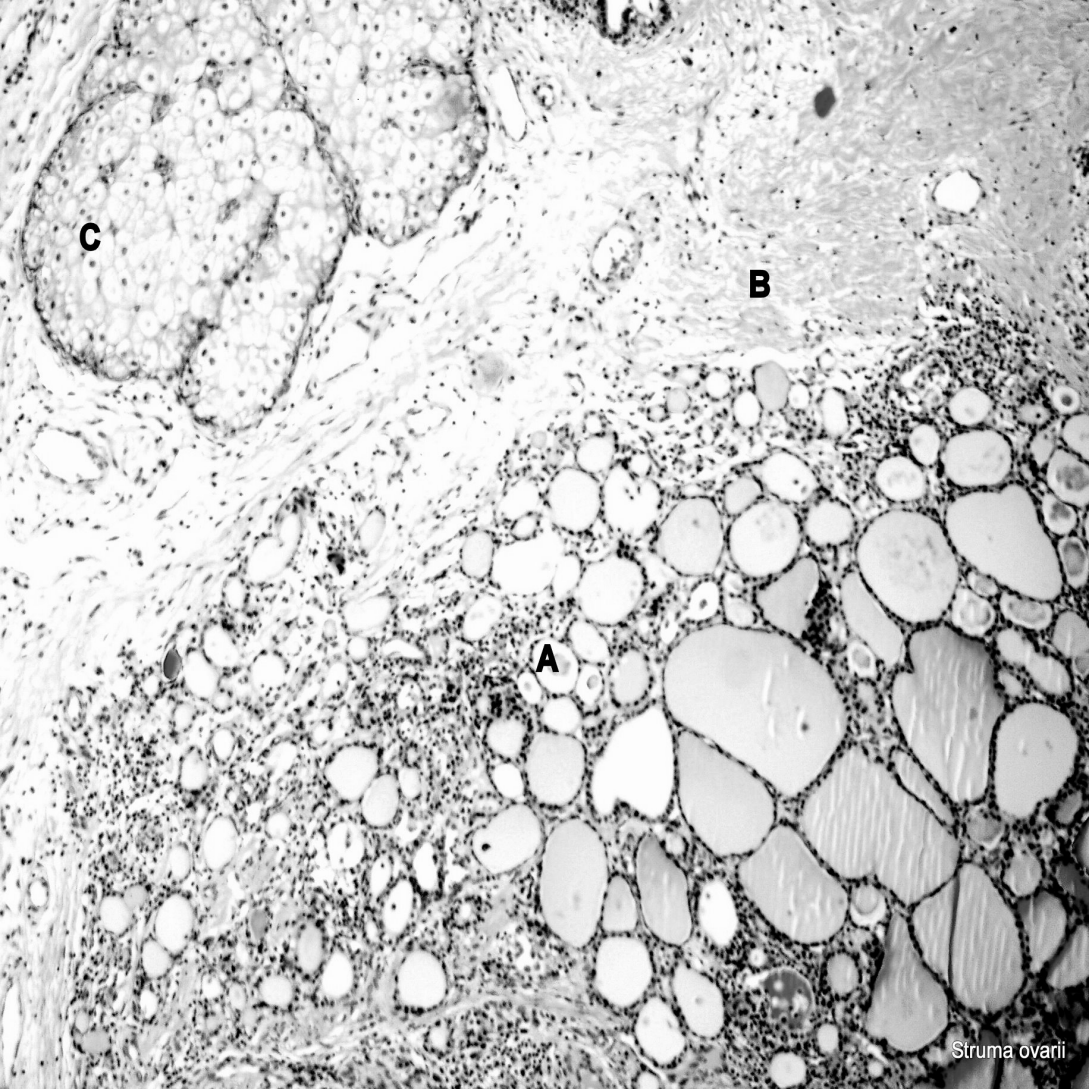
**Struma ovarii (hematoxylin and eosin stain ×100)**. **(A) **Thyroid follicles surrounded by lymphocytes; **(B) **strumal component; **(C) **adipocyte.

A 17-year-old Caucasian female patient had presented at an emergency department with acute abdominal pain. Abdominal ultrasonography revealed a hyperdense cystic mass measuring 20 cm in diameter which included solid components located on the right side and extending to the umbilical level, which was confirmed by abdominal computed tomography (CT). At that time, she underwent emergency abdominal surgery, in which torsion of her right ovary, and the mass arising from it, was seen. A frozen section indicated benign tissue, and the operation was completed with the removal of a 1600-g tumoral mass and a salpingo-oophorectomy on the right. A postoperative pathologic examination had revealed a benign cystic struma ovarii, confirmed by immunohistochemical staining for thyroglobulin. The specimen was remarkable for lymphocytes and lymphoid follicles dispersed among the thyroid follicles, which were consistent with lymphocytic thyroiditis (Figure 1). Thyroid function tests, which had been performed after the urgent operation, revealed subclinical hyperthyroidism (Table 1).

**Table 1:** Laboratory findings and thyroid ultrasonographic features of the patient

	Normal range	Time after surgery			
		
		Three days	Two months	Six months	One year
**TSH (mIU/mL)**	0.27-4.2	0.001	0.004	0.28	2.87
**free-T_4 _(ng/dL)**	0.74-1.52	1.32	1.16	0.86	1.42
**free-T_3 _(pg/mL)**	2.3-4.2	3.6	3.2	3.1	3.68
**Anti-TPO (IU/mL)**	10-115	-	565.3	468.2	286.7
**Anti-TG (U/mL)**	5-60	-	465.7	350.6	139.5
**Thyroid volume^a ^(mm^3^)**	-	-	9905.62	9855.41	9818.8
**Isthmus thickness (mm)**	-	-	3.2	3.3	3.2
**Parenchymal echogenicity**	-	-	Mildly reduced	Mildly reduced	Mildly reduced

^a^Thyroid volume = (right lobe (a×b×c) ml ×0.502) + (left lobe (a×b×c) ml ×0.502)/2

Anti-TG: antithyroglobulin; anti-TPO: antithyroid peroxidase; fT_3_: free triiodothyronine; fT_4_: free thyroxine; TSH: thyroid stimulating hormone.

Two months after surgery, the woman was referred to our clinic with persistent subclinical hyperthyroidism. A thyroid ultrasound, scintigraphic imaging of her thyroid gland with Tc-99 m pertechnetate and iodine-131 whole body scanning were performed. The thyroid ultrasound demonstrated a normal thyroid gland in size and mildly heterogenic parenchymal echogenicity. Functioning ectopic thyroid tissue was not seen in her inguinal or pelvic sections in the whole body scan, but condensed accumulation was observed in her thyroid region, as expected. The thyroid scintigraphy showed condensed focal accumulation in the left thyroid lobe, while other sides of the left lobe remained mildly suppressed and the right lobe was homogenous. Autoimmune thyroiditis was thought to be responsible for this focal condensation; this supposition was later supported by laboratory investigations, which showed elevated levels of serum antithyroglobulin (465.7 IU/mL; normal range, 5 U/mL to 60 U/ml) and antithyroid peroxidase (565.3 IU/mL; normal range, 10 IU/mL to 115 IU/ml), measured by electrochemiluminescence immunoassay with a commercially available kit (Immulite 2000, Bio DPC, Los Angeles, CA, USA). A radioiodine uptake test was performed with 20 mCi I-131 and the results revealed a decreased uptake, by 16% after four hours, and 27% after 24 hours. This result showed that the thyroid gland was also affected, which decreased the iodine uptake, presumably due to the autoimmune thyroiditis that originated within the ectopic thyroid tissue. Because our patient had been symptomatic for one month, she was started on propylthiouracil (PTU), after confirming the absence of any residue or recurrence with abdominal magnetic resonance imaging. After one month of treatment her serum thyroid stimulating hormone level normalized and her thyroid antibody levels decreased, and so PTU was stopped.

## Discussion

Struma ovarii comprise approximately 1% of all ovarian tumors and 2% to 5% of all ovarian teratomas. They are composed of mature thyroid tissue -which mostly (approximately 95%) has a benign nature- occupying more than 50% of the mass [1]. Thyroid tissue in the teratoma can exhibit all histological and pathological patterns of normal thyroid epithelium, such as an adenoma or as papillary or follicular carcinoma. It may also organize in solid, embryonal or pseudotubular patterns [3]. Immunohistochemical staining for thyroglobulin may be required to identify the cells' origin. Thyrotoxicosis originating from a struma ovarii is a rare condition seen in 5% to 10% of patients, mostly resulting from adenomas and papillary carcinomas [2]. Some investigators reported autoimmune thyroiditis (Graves' disease) of struma ovarii to be the cause of thyrotoxicosis. Teale and colleagues demonstrated the presence of thyrotropin receptors on a struma ovarii in a patient with co-existing Graves' disease who had undergone remission after removal of her ovary [4]. They suggested that circulating thyrotropin receptor antibodies could stimulate the tumor and cause thyrotoxicosis, confirming a case reported by Kung *et al*. [5]. There are other case reports detailing the co-existence of Graves' disease and struma ovarii in thyrotoxic patients, however Hashimoto's thyroiditis of a struma ovarii was described in only a few case reports. Morrissey speculated that a struma ovarii resulted in antithyroidal antibody formation and thus might have caused secondary thyroiditis in a patient with thyrotoxicosis, who had become euthyroid after surgery [6]. Bonadio presented the case of a patient with Hashimoto's thyroiditis in a struma ovarii, diagnosed due to the presence of antithyroid antibodies in the absence of any symptoms and signs of thyroid disease [7]. Doldi *et al*. reported another patient diagnosed with Hashimoto's thyroiditis in a malignant struma ovarii based on positive antimicrosomal and antithyroglobulin antibodies [8]. On the other hand, benign thyroid tissue, which can spread to the peritoneal cavity (peritoneal strumosis), has been reported to cause thyrotoxicosis in a patient with a struma ovarii. We diagnosed Hashimoto's thyroiditis in a struma ovarii, not only based on the presence of antithyroid antibodies, but also confirmed by the histopathologic examination. Although our patient had neither history of a thyroidal disease nor related symptoms before, we cannot speculate that thyroiditis in the struma ovarii had first started the clinical progress, unlike in the case described by Morrissey. Besides, we do not know the status of her thyroid function and antibodies prior to surgery. Symptoms related to thyroiditis became overt within two months of her surgery, possibly triggered by the administration of the contrast agent for tomography. Struma ovarii can be suspected in women with thyrotoxicosis but who do not have a goiter and who have minimal or absent thyroid uptake of radioiodine. However, struma ovarii are rare among other more likely causes of these findings, such as exogenous thyroid hormone administration and thyroiditis. The diagnosis can be made by radioiodine imaging of the pelvis through whole body scanning, which would show the increased accumulation in the ovary [3]. Increased thyroglobulin levels can support a diagnosis of struma ovarii if antithyroglobulin antibodies are absent. The serum thyroxin and triiodothyronine ratio and a low thyroglobulin level can be considered in thyrotoxicosis of exogenous intake. Postoperative I-131 total body scanning and serum thyroglobulin monitoring is recommended for the management and follow-up of patients with malignant struma ovarii for residue, recurrence or metastasis. However, the sensitivity of I-131 scanning is very low for the detection of residual or recurrent malignant tissue, even after total ablation of the thyroid gland [9]. Besides, thyroglobulin cannot be considered as a tumor marker in patients whose thyroid gland has not been totally ablated and/or who are positive for the antithyroglobulin antibody. Therefore, most authors recommend the removal of the thyroid gland for follow-up of patients with a malignant struma [10]. Metastasis is not characteristic for benign struma ovarii but recurrence is a rare condition. Our patient's persistent thyrotoxicosis alerted us firstly to look for a residue and then for recurrence, maybe on the other ovary because of the normal appearance of her thyroid gland. Whole body scanning was not a reliable diagnostic test because she had a functioning thyroid gland, nor was her thyroglobulin level because of antithyroglobulin positivity. Besides, we found thyroiditis in a struma ovarii. A thyroidectomy was not considered given her young age. We performed abdominal magnetic resonance imaging and ultrasound to exclude residue or recurrence.

## Conclusion

We present the case of this patient because we found Hashimoto's thyroiditis in struma ovarii and we wanted to emphasize the difficulties in management of this rare condition. There is a paucity of data in the past literature regarding the optimal follow-up modality for such patients; our findings may be auxiliary to other clinicians.

## Consent

Written informed consent was obtained from our patient and her legal guardian for publication of this case report and any accompanying images. A copy of the written consent is available for review by the Editor-in-Chief of this journal.

## Competing interests

The authors declare that they have no competing interests.

## Authors' contributions

NCB is the corresponding and primary author of this case report. BK and EÇÖ analyzed and interpreted the patient's data. EÇ and TD were contributors for writing the manuscript's discussion. All authors read and approved the final manuscript.
